# Advancing Reverse Electrowetting‐on‐Dielectric from Planar to Rough Surface Electrodes for High Power Density Energy Harvesting

**DOI:** 10.1002/ente.202100867

**Published:** 2022-01-07

**Authors:** Pashupati R. Adhikari, Adnan B. Patwary, Karthik Kakaraparty, Avinash Gunti, Russell C. Reid, Ifana Mahbub

**Affiliations:** ^1^ Department of Mechanical Engineering University of North Texas Denton TX 76207 USA; ^2^ Department of Electrical Engineering University of North Texas Denton TX 76201 USA; ^3^ Department of Engineering Dixie State University St. George UT 84770 USA

**Keywords:** high surface areas, rough electrodes, low-frequency motion energy harvesting, mathematical modeling, reverse electrowetting-on-dielectric, wearable motion sensors, zero applied bias

## Abstract

Reverse electrowetting‐on‐dielectric (REWOD)‐based energy harvesting has been studied over the last decade as a novel technique of harvesting energy by actuating liquid droplet(s) utilizing applied mechanical modulation. Much prior research in REWOD has relied on planar electrodes, which by its geometry possess a limited surface area. In addition, most of the prior REWOD works have applied a high bias voltage to enhance the output power that compromises the concept of self‐powering wearable motion sensors in human health monitoring applications. In order to enhance the REWOD power density resulting from an increased electrode–electrolyte interfacial area, high surface area electrodes are required. Herein, electrical and multiphysics‐based modeling approaches of REWOD energy harvester using structured rough surface electrodes are presented. By enhancing the overall available surface area, an increase in the overall capacitance is achieved. COMSOL and MATLAB‐based models are also developed, and the empirical results are compared with the models to validate the performance. Root mean square (RMS) power density is calculated using the RMS voltage across an optimal load impedance. For the proposed rough electrode REWOD energy harvester, maximum power density of 3.18 μW cm^−2^ is achieved at 5 Hz frequency, which is ≈4 times higher than that of the planar electrodes.

## Graphical abstract

Low‐frequency motion (1–5 Hz) energy harvesting based on reverse electrowetting‐on‐dielectric is presented using structured high surface area rough electrodes. The energy harvester is capable of generating up to 3.18 μW cm^−2^ of power density without external bias voltage, making the harvester suitable to power up wearable motion sensors. Empirical results are validated and supported with developed mathematical models.

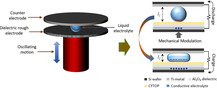

## 1 Introduction

The phenomenon of the apparent surface tension of a liquid on a solid surface changing with applied electric field is known as electrowetting and has long been studied. Electrowetting that takes place on a dielectrically insulated surface is further known as electrowetting‐on‐dielectric (EWOD). EWOD on a superhydrophobic surface has various applications and is a topic of increasing interest.^[^ ^1^, ^2^, ^3^, ^4^, ^5^ ^]^ Krupenkin et al., a decade ago, put forward a novel work reversing the idea of EWOD, and demonstrated electrostatic energy generation from mechanical motion of a liquid droplet. This phenomenon is known as reverse electrowetting‐on‐dielectric (REWOD) energy harvesting.^[^ ^6^ ^]^ Liquid droplet‐based energy harvesting using reverse electrowetting effects has gained momentum in the recent years.^[^ ^7^, ^8^ ^]^ Unlike many other energy harvesting technologies, REWOD has been demonstrated to operate efficiently at a low mechanical frequency range because of its independence from the resonance of the solid structures.^[^ ^9^, ^10^ ^]^ The REWOD mechanism is the opposite of EWOD, where an applied voltage results in a mechanical motion of liquid droplet(s). In REWOD, an applied mechanical force results in a voltage due to an increase in the electrical capacitance.

**Figure 1:**
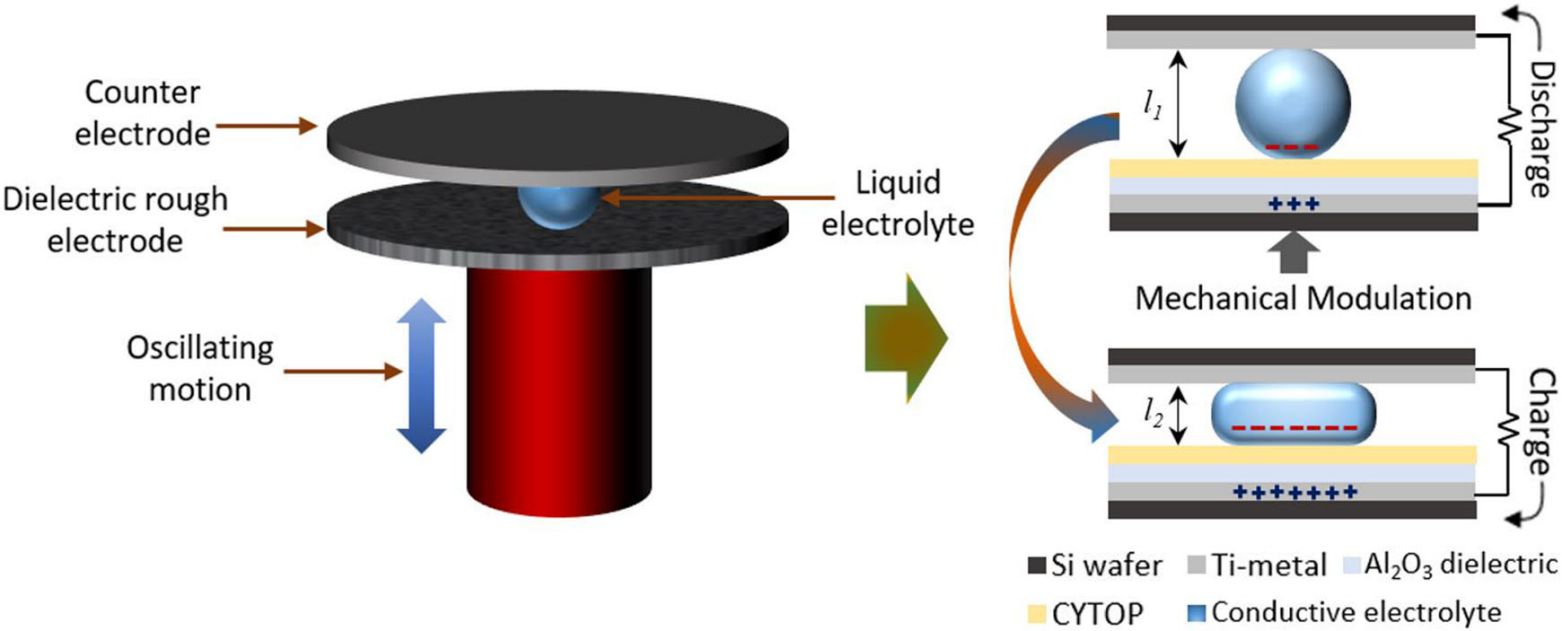
Working principle of REWOD energy harvesting without any applied bias voltage. Maximum mechanical displacement of electrodes during modulation, Δ*l* is given as: Δ*l = l*_1_ *−l*_2_.

The working mechanism of REWOD energy harvesting is illustrated in **Figure** **1**. The configuration shown in the figure includes a top electrode, which is coated with a metal layer that acts as a current collector and a bottom electrode, which is first coated with a metal layer for electrical conduction, then with a metal oxide dielectric layer (e.g., Al_2_O_3_ or SiO_2_), and finally with an additional layer of fluoropolymer (e.g., Teflon or CYTOP) for surface hydrophobicity. A spin‐coated thin layer of fluoropolymer CYTOP has been used for hydrophobic applications in prior works.^[^ ^11^, ^12^ ^]^ Apart from hydrophobicity, fluoropolymer CYTOP layer also acts as an electret, which is a dielectric with quasipermanent charges on its surface sustaining high surface charge density. CYTOP is a well‐known electret and even without using a corona discharge, the inherent voltage of the CYTOP could be enhanced through the use of charge injection during electrowetting. CYTOP as a precharged electret has been successfully used in other energy harvesting technologies.^[^ ^13^ ^]^ An electrolyte is placed in between the electrodes, which upon oscillation due to a force from the external mechanical input generates an AC current. The AC current generation in REWOD depends on several parameters such as dielectric material, surface charge density, surface hydrophobicity, modulation frequency, and electrode–electrolyte interfacial area. In this work, the bottom electrode is made to be rough by implementing reactive ion etching (RIE) to increase the total available surface area. Increasing surface area results in a higher electrode–electrolyte interfacial area which is proportional to the capacitance, and hence the power density output.

The capacitance at the electrode–electrolyte interface in REWOD is the result of the capacitance from the electrical double layer (EDL) at both the uninsulated top metal electrode and the insulted bottom electrode. However, as the capacitance at the uninsulated electrode is much larger ($C=\varepsilon A/d,d_{\text{bare}}\ll d_{\text{insulated}}$), it is therefore neglected in this work because its contribution to the total capacitance is negligible when in series with the capacitance at the insulated electrode. The bottom electrode (insulated) has a certain dielectric thickness, *d*, which is ≈200 nm in total and the top electrode (uninsulated) has zero dielectric thickness and only possesses the thickness of the EDL that is formed at the interface. Compared to dielectric thickness, *d* of the bottom electrode (≈200 nm), the thickness of the EDL formed at the deionized (DI) water–Ti interface is significantly lower. It has been reported that the EDL thickness can range from 0.1 to 2.5 nm depending on the metal surface and the electrolyte concentration.^[^ ^14^ ^]^ Accordingly, the capacitance at the uninsulated electrode–electrolyte interface is much higher (*C = εA/d*) compared to that at the insulated electrode–electrolyte interface. As these two electrical double layer capacitances (EDLCs) are in series, the higher capacitance from the uninsulated electrode–electrolyte interface has negligible contribution to the total capacitance and is therefore neglected.

Many existing energy harvesting technologies that were proposed before REWOD have their own significance in specific applications.^[^ ^15^, ^16^, ^17^, ^18^, ^19^ ^]^ However, for low‐frequency motion activities, most if not all fail to perform or show very poor performance. REWOD has been demonstrated to operate efficiently at a low‐frequency (1–5 Hz) range.^[^ ^20^, ^21^, ^22^, ^23^ ^]^ Most REWOD energy harvesting research to date has solely relied on harvesting higher energy output by applying a range of bias voltages from an external source, which directly compromises the idea of self‐powering wearable and implantable electronics.^[^ ^24^, ^25^ ^]^ Our work in REWOD energy harvesting thus far has been focused on harvesting sufficient energy without any external bias source to power up wearable and implantable electronics for human health monitoring.^[^ ^20^, ^21^, ^26^, ^27^ ^]^ As a novel approach, the proposed work contributes to REWOD energy harvesting by enhancing power density output as a result of enhanced surface area because of the implementation of structured rough electrodes.

In REWOD, the wetting and dewetting phenomenon on a structured surface, such as the one used in this work, is significantly different than those on a flat surface. The fabrication steps involved in this work (plasma etching, physical vapor deposition of metal and dielectric layers, and spin coating of CYTOP layer) make the surface slightly rougher and introduce the possibility of air being trapped under the electrolyte droplet once introduced (the Cassie–Baxter state). However, due to the relatively small aspect ratio of the surface features (feature height divided by distance between features), the Cassie state is not the dominant state.^[^ ^28^ ^]^ Rather, the Wenzel state (electrolyte penetrates into the pores over a very short period of time) likely occurs for a complete wetting of the structured rough surface in this work.

All of the previous research on REWOD energy harvesting successfully demonstrated AC current generation. It is, however, apparent that these works used bias voltage on planar electrodes and did not attempt to enhance power density by increasing the surface area. As wearable and implantable electronics have become miniaturized over the years, the space available for integrating an energy harvester into the electronics is limited.^[^ ^29^, ^30^, ^31^ ^]^ Reducing the planar area while enhancing the total available surface area either by roughening the surface or by using porous electrodes would be a solution to this problem. Apart from the zero applied‐bias‐voltage approach of the proposed work, our work focuses on increasing the surface area by using the structured rough electrodes. The present work is a novel path toward increasing the REWOD power density which would be useful for miniaturized wearable and implantable electronics. Theoretical results derived from Multiphysics COMSOL and MATLAB‐based models are also included in the present work. These models are validated using measurement data to further demonstrate the feasibility of the REWOD energy harvesting as a source of power in wearable and implantable electronics.

## 2 Experimental Section

### 2.1 Material Fabrication

**Figure 2:**
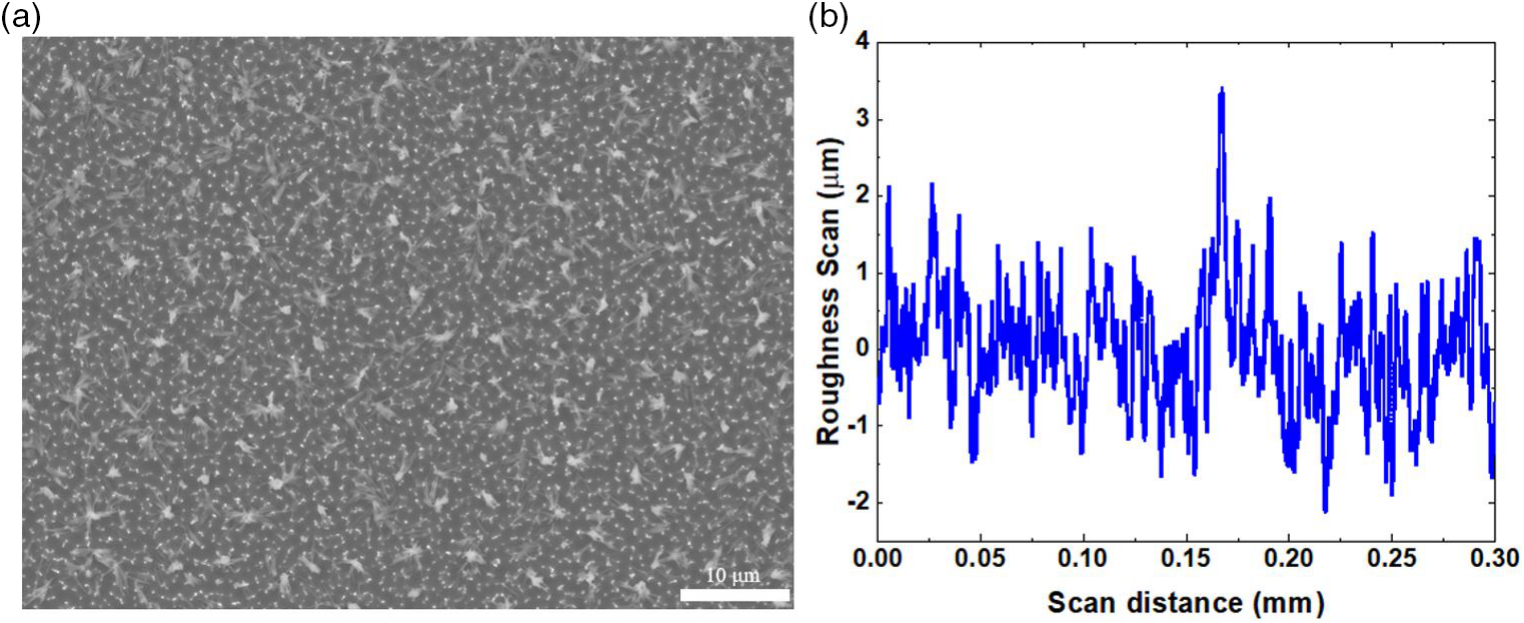
a) FE‐SEM image of the rough electrode after RIE. b) A profilometer scan of the rough electrode to estimate its roughness. The peaks and valleys of the rough surface average ≈5–6 μm of roughness (the distance is taken from the highest peak to the lowest valley).

Two dissimilar electrodes were fabricated using (single side polished) highly doped P‐type silicon wafers with a diameter of 100 mm and a thickness of 0.38 mm (University Wafers Inc.). In order to create rough electrodes using the Si wafer, RIE technique was adopted (AGS RIE MPS‐150). A single wafer was plasma etched under the presence of carbon tetrafluoride (CF_4_) at a flow rate of 40 sccm, 200 mTorr of pressure, and a DC power of 500 W for a total of 45 min at an increment of 15 min for 3 times. Etching progress on the wafer was visually checked after each of the 3 cycles to ensure etching consistency. Once etching was completed, the wafer was diamond cut into four equal parts (quarters) and samples were subjected to further fabrication processes as described below. A field emission scanning electron microscope (FE‐SEM) image of one of the etched samples is shown in **Figure** **2**a. A profilometer scan using Alpha‐Step D‐300 Stylus Profiler (KLA Corporation) was performed to examine the heights and depths of the peaks and valleys of the etched wafer. One of the scans is shown in Figure 2b, showing a peak‐to‐peak height between peaks and valleys of ≈5–6 μm. Planar electrodes were fabricated using a similar approach to that of our prior work on REWOD energy harvesting.^[^ ^20^ ^]^

Top and bottom electrodes (Figure 1) are referred to as dissimilar in this work because the two electrodes are different in their surface roughness as well as in the coatings that were applied. Both wafer electrodes were first coated with a ≈200 nm‐thick Ti adhesion layer to create a metal contact. Before the dielectric material deposition over the Ti layer on the rough electrode, a small portion of the wafer was covered with a Kapton tape to block the dielectric insulation, which was later removed to enable current conduction. Subsequently, ≈150 nm of dielectric material (Al_2_O_3_) was deposited. The Ti and dielectric materials were deposited using a NEE‐400 dual e‐beam evaporator (Nanomaster Inc.). After each deposition, thicknesses were verified using the profilometer.

After a successful deposition of the desired thickness of Al_2_O_3_, the rough bottom electrodes were deposited with an additional layer of hydrophobic material. A fluoropolymer, CYTOP (CTL‐809M), and its solvent (CT‐Solv. 180), both purchased from AGC Chemicals Company, were mixed together in a volumetric ratio of 1:3. The solution was spin‐coated on the wafers over the dielectric layer. Spin coating was performed at 600 rpm for 5 s (spread cycle), and then 3000 rpm for 50 s (spin cycle). The samples were dried at room temperature for 15 min, pre‐baked for 30 min at 80 °C, and final‐baked for 60 min at 185 °C to ensure complete evaporation of the solvent.

### 2.2 Measurement Setup

**Figure 3:**
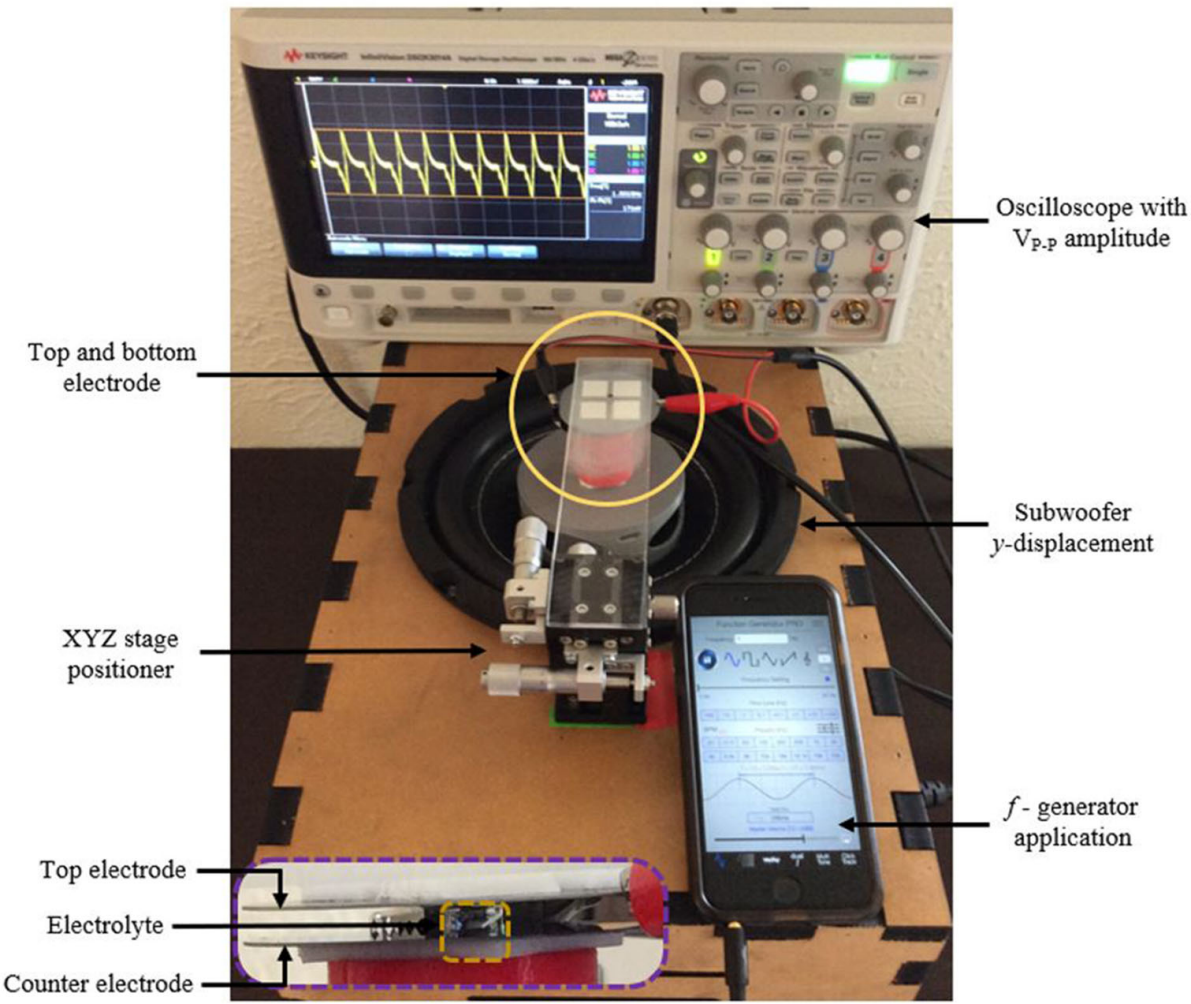
Measurement setup for REWOD energy harvesting. Electrolyte modulation between the two dissimilar electrodes is shown in the inset with the electrolyte drop contained within a yellow dashed line.

The measurement setup for the AC current and power generation measurement is illustrated in **Figure** **3**. A similar measurement setup has been used in our prior works involving REWOD energy harvesting.^[^ ^20^, ^23^, ^26^, ^27^ ^]^ It consists of an XYZ positioner stage with a long and lightweight acrylic beam attached to it. The beam is used to hold the top electrode stationary. The *y*‐axis of the XYZ positioner is set to a distance of 4 mm from the bottom electrode before the start of the modulation. Input oscillations were applied using a custom‐built subwoofer system that can be controlled with a signal generating app (Audio Function Generator PRO). This application works almost the same way as an actual function generator, except it excites the subwoofer in vertical mechanical displacement to a desired amplitude. Similar custom‐made systems have been reported in prior energy harvesting research.^[^ ^32^, ^33^ ^]^ During the electrolyte modulation between the electrodes, the generated AC voltage was measured using an oscilloscope (Keysight InfiniiVision DSOX3014A), AC current was measured using a Keithley 2400 Sourcemeter, and the measurement results were acquired using Keithley data acquisition software, Kickstart 2.0. The resistance and capacitance measurements were performed using an impedance and electrochemical front end (AD5940) by Analog Devices.

**Figure 4:**
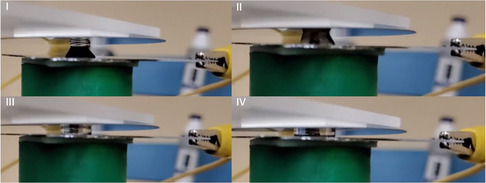
Still video images of four different stages of the electrolyte being modulated between the electrodes.

**Figure** **4** shows four different stages of the electrolyte being modulated within an electrode gap ranging from 4 to 1.5 mm (resulting in 2.5 mm of electrode displacement). At 4 mm distance, the electrolyte geometrically forms almost a conical shape, implying that the interaction between the bottom electrode and the electrolyte is stronger than that between the top electrode and the electrolyte. The stronger interaction between the bottom electrode and the electrolyte is attributed to gravitational force acting on the electrolyte. For an electrolyte droplet size as large as 50 μL, the gravitational force could be significant. In order to investigate the effect of the gravitational force and also to determine whether the surface tension dominates the gravitational force, the Bond number (Bo) was calculated. Bo is a dimensionless number and is given by Equation (1). If Bo is much less than one, then that implies that the surface tension to some extent dominates the gravitational force.

(1) $${\text{Bo}}_{}=\frac{\Delta \rho gR^{2}}{\gamma }$$

where Δ*ρ* is the difference in density of two phases, the gas and the liquid phases, *g* is the acceleration due to gravity, *R* is the characteristic length of the liquid droplet (the radius), and *γ* is the surface tension. Considering close to a perfect sphere and the maximum distance between the top and the bottom electrode, the characteristic length of the 50 μL DI water droplet was mathematically approximated to be 2.5 mm. Note that laboratory grade DI water was used as an electrolyte in this work. Using the standard density values of air and DI water, 1.225 and 999.84 kg m^−3^, respectively, and the surface tension of 0.072 N m^−1^ for DI water from Han et al., the Bo was calculated to be 0.85, which is very close to one showing that there is a significant effect of gravity on the electrolyte droplet in addition to the surface tension.^[^ ^34^ ^]^ Therefore, the shape of the electrolyte at stage I in Figure 4 is conical that gradually diminishes and translates into stage IV through stages II and III in the figure to a perfectly cylindrical disc. Ultimately, we are only interested in the maximum capacitance that occurs when the electrodes are closest together and the electrolyte forms a perfect cylindrical disk. In our prior work on planar electrodes, it was determined that the maximum electrode–electrolyte interfacial area was 0.33 cm^2^ at 1.5 mm electrode distance and 50 μL electrolyte.^[^ ^20^ ^]^ We have used the same interfacial area in this work given that the electrolyte volume as well the electrode displacement have remained the same. The maximum electrode–electrolyte interfacial area is used to determine the power density.

## 3 Theoretical Modeling

### 3.1 Electrical Equivalent Circuit Modeling

**Figure 5:**
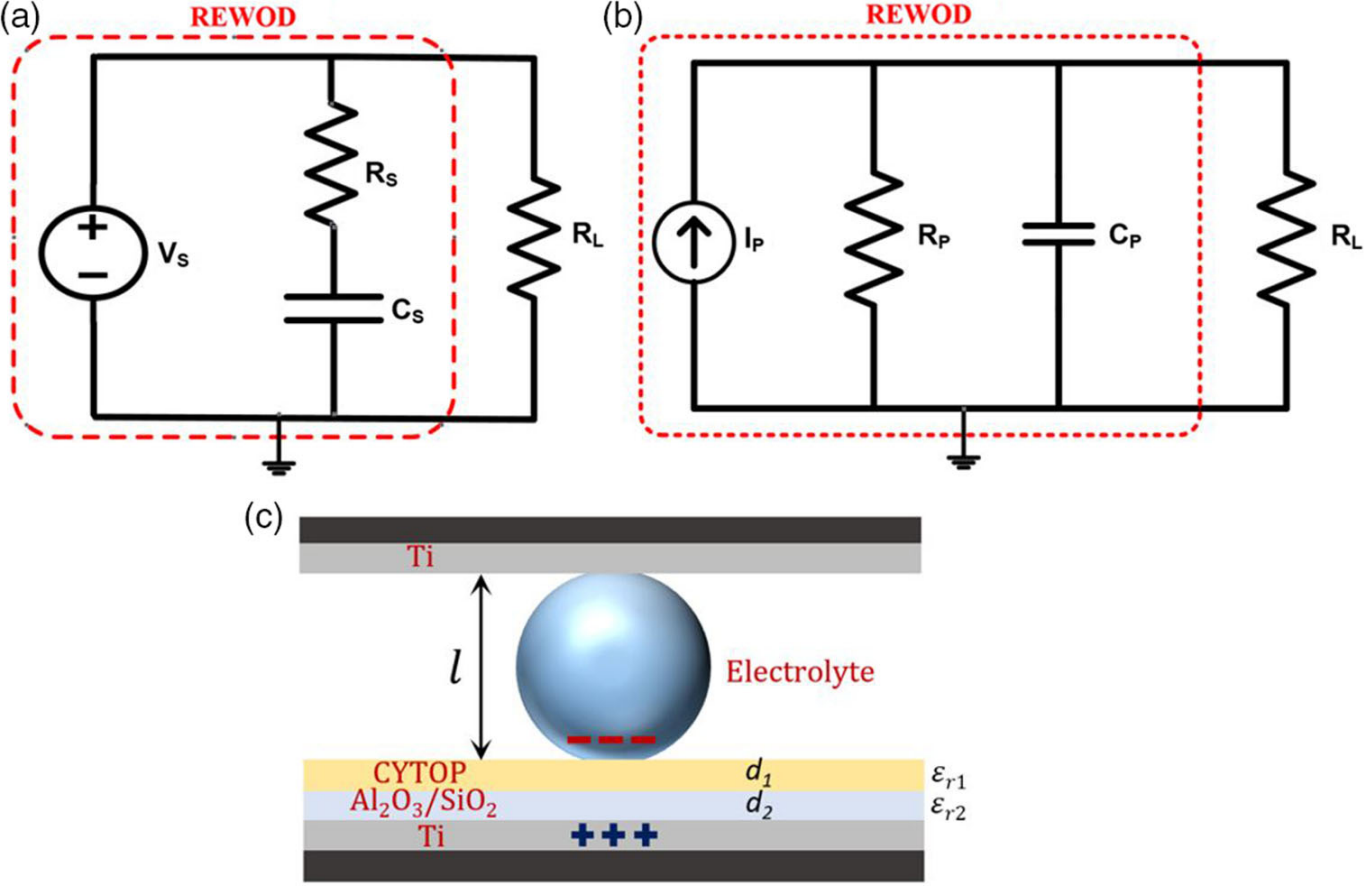
a) Lumped element model of a REWOD energy harvester in series configuration. b) Lumped element model of a REWOD energy harvester in parallel configuration. c) Electrolyte bridge between dielectric and conductive layers, showing relevant dielectric constants (*ε*_r1_ and *ε*_r2_) of the dielectric layers and their thicknesses (*d*_1_ and *d*_2_).

The AC current generation associated with electrolyte modulation between the electrodes depends on the change in the capacitance between them. Theoretically, REWOD can be approximately modeled using lumped element‐based electrical circuit components either in a series configuration as shown in **Figure** **5**a, or a parallel configuration as shown in Figure 5b. The accompanying Figure 5c shows the electrolyte bridge between the dielectric and the conductive layers showing relevant dielectric constants for the liquid electrolyte of thickness *l* (the gap between the top and bottom electrodes) and dielectric layers of total thickness *d (d = d*_1_ *+ d*_2_). As discussed earlier, the dielectric electrode was fabricated by dry etching such that the surface is rough and available surface area is enhanced to increase the capacitance density (F/cm^2^). The model includes a resistor *R*_P_, a capacitor *C*_P_, and a current source *I*_P_ in parallel. *I*_P_ represents the generated AC current which is the rate of change of generated charge on the REWOD electrodes. *C*_P_ acts as a variable capacitor that changes periodically during the electrolyte modulation, while electrical resistance, *R*_P_, occurs across the electrodes due to the electrical conductivity and thicknesses of the electrolyte, dielectric, and conductive layers along with the electrode–electrolyte interfacial area. Parallel arrangement of the capacitor and resistor elements in the circuit model (Figure 5b) as opposed to a series arrangement (Figure 5a) eliminates the complexity of requiring two different voltages across the resistor and capacitor in the modeling. Most piezoelectric and pyroelectric energy harvesting models also assume a similar parallel arrangement while modeling the electrical equivalent circuit.^[^ ^35^, ^36^ ^]^ Accordingly, we have adopted the parallel arrangements of the resistor *R*_P_, the capacitor *C*_P_, and current source *I*_P_.

### 3.2 MATLAB Model

The REWOD‐based energy harvester is modeled in MATLAB in order to gain additional insights into factors that affect performance, specifically the capacitance and the REWOD resistance. Prior works also suggest that the pressure variation and the contact angles between the electrolyte and the electrodes can be analyzed accurately through a MATLAB model.^[^ ^37^ ^]^ The FE‐SEM image of the rough surface electrode and the profilometer data from Figure 2b were considered in order to analyze and estimate the roughness of the surface in the implementation of the MATLAB model of the REWOD energy harvester. The primary goal of the MATLAB modeling of the REWOD setup is to generate the capacitance, current, and voltage values, and then to perform a comparative analysis based on the experimentally measured data, which, in turn, gives clear insights into the specific factors that affect the performance of the REWOD systems.

**Figure 6:**
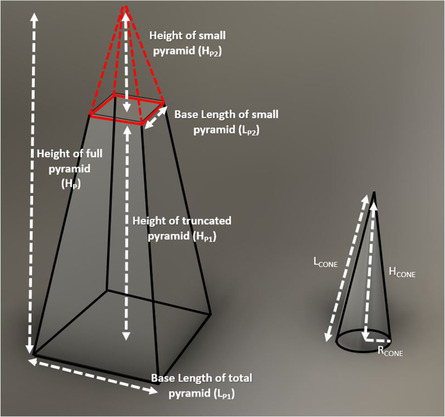
Primitive blocks used to estimate surface area in MATLAB and COMSOL models.

The rough electrode surface has been modeled by approximating it with truncated pyramids and regular cones as shown in **Figure** **6**. The features that are present in the FE‐SEM image shown in Figure 2a, when simplified, resembled truncated pyramids and cones, where the larger white areas are considered the truncated pyramids and the small white dots which represent small peaks are considered as cones. From the SEM image, the total number of cones and truncated pyramids within a planar area of 118.75 μm^2^ (the approximate area of the SEM image shown in Figure 2a) were approximated to be 91 and 5, respectively. In reference to the scan data given in Figure 2b, the specific model parameters such as number of peaks, average height of the peaks, and the surface area are summarized in **Table** **1**. The geometric parameters defined in Table 1 are illustrated in Figure 6. As we increase the frequency of modulation during experiments, the electrolyte impingement takes place and as a result the number of cones and truncated pyramids covered by the electrolyte increases, which leads to increasing electrode–electrolyte interfacial area. The lateral surface area of the truncated pyramid, *A*_P_, is given by Equation (2)

(2) $$A_{P}=2L_{p1}\sqrt{\frac{L_{p1}{}^{2}}{4}+H_{p1}{}^{2}}-2L_{p2}\sqrt{\frac{L_{p2}{}^{2}}{4}+H_{p2}{}^{2}}$$

where *L*_p2_ and *L*_p1_ are the base lengths and *H*_p2_ and *H*_p1_ are the heights of the small pyramid portion at the top that is eliminated and the truncated pyramid, respectively. All the respective values are tabulated in Table 1. Additionally, the lateral surface area of the cone, *A*_C_, is given by Equation (3)

(3) $$A_{C}=\pi r_{c}\sqrt{h_{c}{}^{2}+r_{c}{}^{2}}$$

where *h*_c_ is the height of cones and *r*_c_ is the base radius of the cones. Equation (4) represents the effective area that has been computed based on specific parameters such as lateral surface area, base area of cones, and truncated pyramids. The model area represents the electrode–electrolyte interfacial area.

(4) $$\text{Effective area}=\text{Planar area}-N_{C}A_{\text{bc}}-N_{P}A_{\text{bp}}+N_{C}A_{C}+N_{P}A_{P}+N_{P}A_{\text{tp}}$$

where *N*_c_ and *N*_p_ are the number of cones and truncated pyramids and *A*_bc_ and *A*_bp_ are the base areas of the cones and truncated pyramids, respectively. *A*_c_ and *A*_p_ are the lateral areas of the cones and the truncated pyramids, respectively. Within the planar area of 118.75 μm^2^, the total rough surface area of 562.815 μm^2^ was determined showing a surface area enhancement of ≈5 times from planar to rough electrode.

**Table 1:** Summary of the specific geometric parameters that define the modeled rough electrode surface

Parameter	Symbol	Numeric value
Number of cones considered within 118.75 μm^2^ area	*N*_C_	≈91
Number of truncated pyramids considered within 118.75 μm^2^ area	*N*_P_	≈5
Average height of cones	*H*_C_	2.75 μm
Average diameter of cones	*D*_C_	0.96 μm
Average height of truncated pyramids	*H*_P_	3.75 μm
Average base length of truncated pyramids	*L*_P1_	2.3 μm
Average side length for the top of truncated pyramids	*L*_P2_	1.2 μm
Lateral surface area of cones	*A*_C_	3.469 μm^2^
Lateral surface area of truncated pyramids	*A*_P_	28.66 μm^2^
Effective rough area determined	*A*_eff_	562.815 μm^2^

A capacitance is generated across the electrodes as a result of the EDL at the interface between the electrolyte and the electrode. Equation (5) shows how the change in capacitance is determined by the changing surface area.

(5) $$\frac{dC\left(t\right)}{dt}=\frac{\varepsilon_{o}\varepsilon_{\text{eff}}}{d_{1}+d_{2}}\frac{dA\left(t\right)}{dt}$$

where *dA(t)/dt* is the change in the surface area with time, and *d*_1_ and *d*_2_ are the thicknesses of the CYTOP layer and the Al_2_O_3_ layers, respectively. The current and voltage values are computed using Equation (6) and (7), respectively. A detailed derivation of the equations is given in Appendix A of the supplementary information.

(6) $$I_{P}(t)=I_{1}(t)+I_{2}(t)+I_{3}(t)$$

(7) $$V_{P}(t)=\frac{V_{S,\text{DC}}\frac{\partial C_{P}\left(t\right)}{\partial t}+C_{p}(t)\frac{\partial V_{S,\text{AC}}\left(t\right)}{\partial t}}{2\pi fC_{P}\left(t\right)}$$

### 3.3 COMSOL Model

In addition to MATLAB, COMSOL Multiphysics was also used to model the capacitance, voltage, and currents of the rough REWOD energy harvester. The built‐in modeling tool and the functions of COMSOL were used to create the 3D model of the rough surface REWOD topology, including each layer of the top and bottom electrodes as well as the liquid electrolyte. After the 3D modeling of each layer, the appropriate material properties were assigned to the layers. The COMSOL model for the REWOD rough surface was developed for a representative circular area with 10 μm diameter resulting in a 78.53 μm^2^ model area. According to the rough surface model presented in the MATLAB model section, there are approximately 91 cones and 5 pyramids in the 118.75 μm^2^ planar area based on the SEM image shown in Figure 2a. Scaling these values, the number of cones and the pyramids within the 78.53 μm^2^ of planar area for the COMSOL model is calculated to be approximately 60 and 3, respectively.

**Figure 7:**
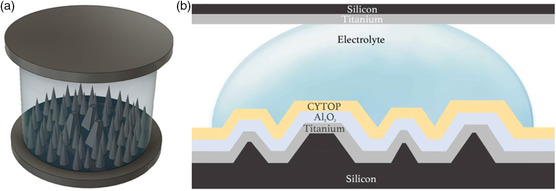
a) 3D COMSOL model portraying rough surface electrode. b) Representative cross‐sectional view of rough electrode model.

**Figure** **7**a depicts the COMSOL rough surface model where the bottom layer is a silicon (Si) wafer with a 0.38 mm thickness. Using the same pyramid and cone density as was used for the MATLAB model, the COMSOL model for the representative unit area has 60 cones and 3 truncated pyramids and therefore the cones and the pyramids were added in the Si layer on the bottom to create the rough surface where the parameter values are taken from Table 1. For the first layer, highly doped silicon material was assigned from the COMSOL materials library, which has a dielectric constant of 4.5 and has a good conductivity. On top of the Si layer is a Ti layer with a thickness of 100 nm, which acts as the metal contact. The Ti layer was modeled as a uniform coating deposited on the pyramids and cones as shown in Figure 7b. For the Ti layer on the bottom, the default Ti metal present in COMSOL was used as the material. On top of the Ti layer, a dielectric layer of 200 nm was placed in the similar manner as the Ti layer. This layer represents a combined layer of Al_2_O_3_ (thickness, *d*_1_ of 150 nm with a dielectric constant, *ε*_r1_ of 9.1), and the CYTOP hydrophobic layer (thickness, *d*_2_ of 50 nm thick with a dielectric constant, *ε*_r2_ of 9.1). The effective dielectric constant, *ε*_eff_, of both the Al_2_O_3_ and the CYTOP layer was calculated to be 4.9 using Equation (8)

(8) $$\varepsilon_{\text{eff}}=\frac{d_{1}+d_{2}}{\frac{d_{1}}{\varepsilon_{r1}}+\frac{d_{2}}{\varepsilon_{r2}}}$$

The materials were assigned manually into COMSOL. On top of the dielectric layer is the electrolyte layer which represents the DI water droplet which works as the electrolyte between the two electrodes of the REWOD model. The dielectric constant of the DI water was assigned to be 86, which was measured using the Keysight N1501A Dielectric Probe Kit and N1500A Materials Measurement Suite. On top of the electrolyte layer, the top electrode is modeled with a Ti layer of 100 nm thickness and 0.38 mm‐thick Si layer. The in‐plane electric current module named “Electrostatics” is chosen to simulate the capacitance and the change in charge for various droplet thicknesses.^[^ ^38^ ^]^ For the COMSOL electrostatic model, the bottom Ti layer was chosen as the ground plane. The top Ti layer and the electrolyte layers were chosen as the terminal plane. Simulations were run for different thicknesses of the droplet (1.5–4 mm) which represents the distance between the electrodes. The model capacitances (*C*_m_) for discrete moments were also calculated for the 78.53 μm^2^ unit area. The overall capacitance (*C*) was calculated using Equation (9)

(9) $$C=\frac{C_{m}A_{\text{eff}}}{A_{m}}$$

where *A*_m_ is the modeled area which is 78.53 μm^2^ and *A*_eff_ is the effective area that is covered by the electrolyte, which varies as the electrolyte is deformed due to the varying distance between the two electrodes. The charge generated by the REWOD model in COMSOL (*Q*m) for the unit area was also obtained from the simulation. The total charge (*Q*) was calculated using Equation (10)

(10) $$Q=\frac{Q_{m}A_{\text{eff}}}{A_{m}}$$

The value of *Q* varies over time (*t*) and therefore by using Equation (11), the electrical current value is obtained. Using the current value, the voltage (*V*) is determined by multiplying the current with the REWOD impedance (*Z*), later discussed under Section 4.2 referencing Equation (12).

(11) $$I=\frac{dQ}{dt}$$

## 4 Results and Discussion

### 4.1 AC Voltage Measurements

**Figure 8:**
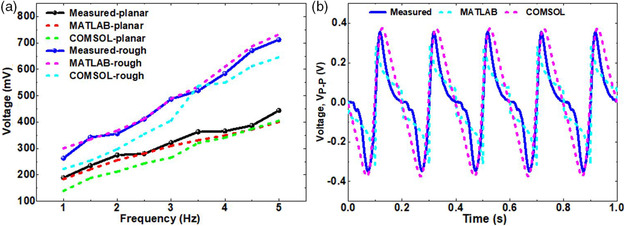
a) Voltage comparison of planar and rough electrodes for measured, MATLAB, and COMSOL models. b) Time scale voltage plot for measured, MATLAB, and COMSOL models at 5 Hz modulation frequency for the first 1 s.

REWOD generated AC voltage was measured for a frequency range of 1–5 Hz with 0.5 Hz step size for both the rough and the planar electrodes using an oscilloscope (Keysight InfiniiVision DSOX3014A). AC voltage increases with increasing frequency, showing almost a linear relationship between the voltage and the frequency. AC peak‐to‐peak voltages for the given frequencies ranged from 189 to 444 mV for the planar electrode and 263–712 mV for the rough electrode. This range of voltages resulted from using a 50 μL droplet of electrolyte (DI water) and 0.33 cm^2^ of electrode–electrolyte interfacial area previously determined.^[^ ^20^ ^]^ Measured voltages at the given frequency range were validated with the results from the MATLAB and COMSOL models. Results show a close agreement between measured and the modeled data. A standard deviation of 19.4 mV from a single frequency at 5.0 Hz using the measured, COMSOL, and MATLAB data for the planar electrode and that of 47.1 mV for the rough electrode validate the agreement. **Figure** **8**a shows a summary result, both measured and modeled, for both planar and rough electrodes. The maximum measured peak‐to‐peak AC voltage at 5 Hz frequency for planar electrode was 444 mV and that for rough electrode was 712 mV, which is a 60% enhancement in AC voltage generation using the rough electrode compared to that of the planar electrode. The AC voltage generation densities are 1.35 and 2.16 V cm^−2^, respectively, for the planar and the rough electrodes. The voltage densities are calculated by dividing the corresponding voltages by the electrode–electrolyte interfacial area of 0.33 cm^2^.

Figure 8b shows a representative AC voltage plot for 1 s of time period at 5.0 Hz frequency involving measured data as well as the data from both the COMSOL and the MATLAB models. The AC voltage signal magnitude and shape for the datasets show a very little difference, indicating a good agreement between the measured and the modeled data. As it can be observed from Figure 8b, the AC signals are not sinusoidal, which could be explained as follows: the contact surface area between the electrode and the electrolyte has a key role in generating the AC voltage. As the wetting properties for the top and the bottom electrode surfaces are different, the contact surface area does not remain the same for the charging and discharging period during the oscillation. As the electrolyte contact area at the top electrode changes at a different rate than that of the bottom electrode, two different *RC* values for charging and discharging occur. As the change in the surface area is not purely sinusoidal, this results in the nonlinearity of the varying capacitance (*C* = *εA*/*d*). Hence, the generated AC voltage does not follow an ideal sinusoidal waveform.

### 4.2 RC Measurements

**Figure 9:**
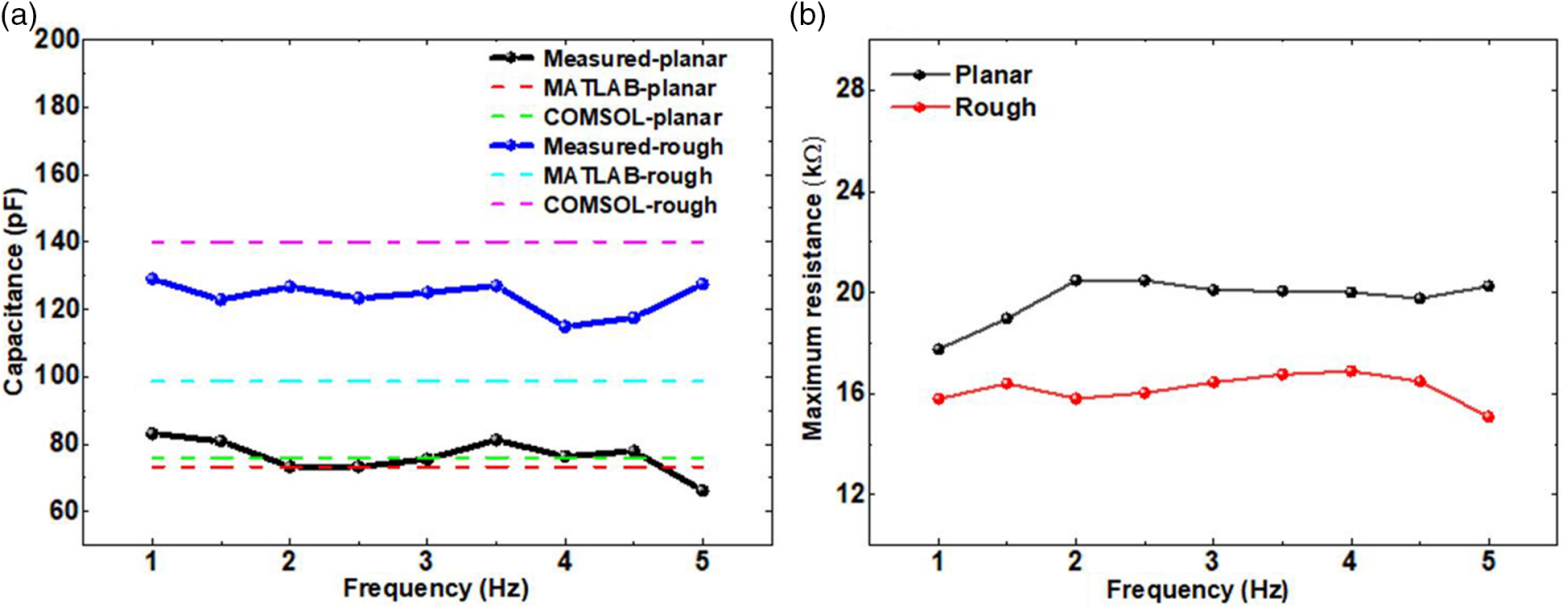
a) Capacitance comparison of planar and rough electrodes for measured, MATLAB, and COMSOL. b) Measured resistance comparison of planar and rough electrodes.

In the modeled REWOD energy harvester with *R*_P_ and *C*_P_ in parallel with *I*_P_ as a current source originating from the REWOD, *C*_P_ acts as a variable capacitor that changes periodically during the electrolyte modulation while electrical resistance, *R*_P_, occurs across the electrodes due to the electrical conductivity and thicknesses of the electrolyte, dielectric, and conductive layers along with the electrode–electrolyte interfacial area. The *R*_P_ and *C*_P_ are measured during modulation using an impedance and electrochemical front end (AD5940) by Analog Devices. This measurement provides the total impedance of the system and phase angle for any given frequency of oscillation. For *R*_P_ and *C*_P_ in parallel, the equivalent impedance is given in Equation (12). The resistance, *R*_P_, is obtained by rearranging the equation for phase angle (Equation 13). The equation for capacitance (Equation 14) results from substituting Equation (13) into Equation (12), and solving for *C*_P_

(12) $$|Z|=\frac{1}{\sqrt{{\left(\frac{1}{R_{P}}\right)}^{2}+{\left(\omega C_{P}\right)}^{2}}}$$

(13) $$R_{P}=\frac{\text{tan}\left(\varphi \right)}{-\omega C_{P}}$$

(14) $$C_{P}=\frac{\text{tan}\left(\varphi \right)}{\omega \left|Z\right|\sqrt{1+{\text{tan}}^{2}\varphi }}$$

where |*Z*| is the absolute impedance, *R*_P_ and *C*_P_ are the resistance and the capacitance of the system, respectively, *φ* is the phase angle, and ω=2πf is the angular frequency of the AC signal where *f* is the applied oscillation frequency. *R*_P_ and *C*_P_ for both planar and the rough electrodes are computed from the measured impedance and phase angle for a modulation frequency range of 1–5 Hz with 0.5 Hz step size with applied reference sinusoidal signal of 10 kHz from the AD5940. **Figure** **9**a shows a capacitance comparison for planar and rough electrodes with respect to frequency for measured and modeled data. Capacitance for the given frequency range is relatively independent of frequency. The capacitances from rough electrodes are higher than those from the planar electrodes by approximately 154% on average. This increase is expected to be much higher (as much as ≈5 times or 500%) because the surface area enhancement per planar area was ≈5 times as described in the COMSOL section of the modeling. Presumably because of the fact that the electrolyte is not entirely in contact with the rough surface due to surface tension effects as the electrolyte moves across the varying peaks and valleys, there is a much smaller electrode–electrolyte interfacial area than expected. Figure 9b shows the measured resistance comparison of planar and rough electrodes. Resistance from rough electrodes is much lower than that from the planar electrodes. Higher electronic conduction due to enhanced rough surface minimizing surface resistance may have attributed to such a low resistance for the rough electrodes compared to the planar electrodes.

### 4.3 AC Current Measurements

**Figure 10:**
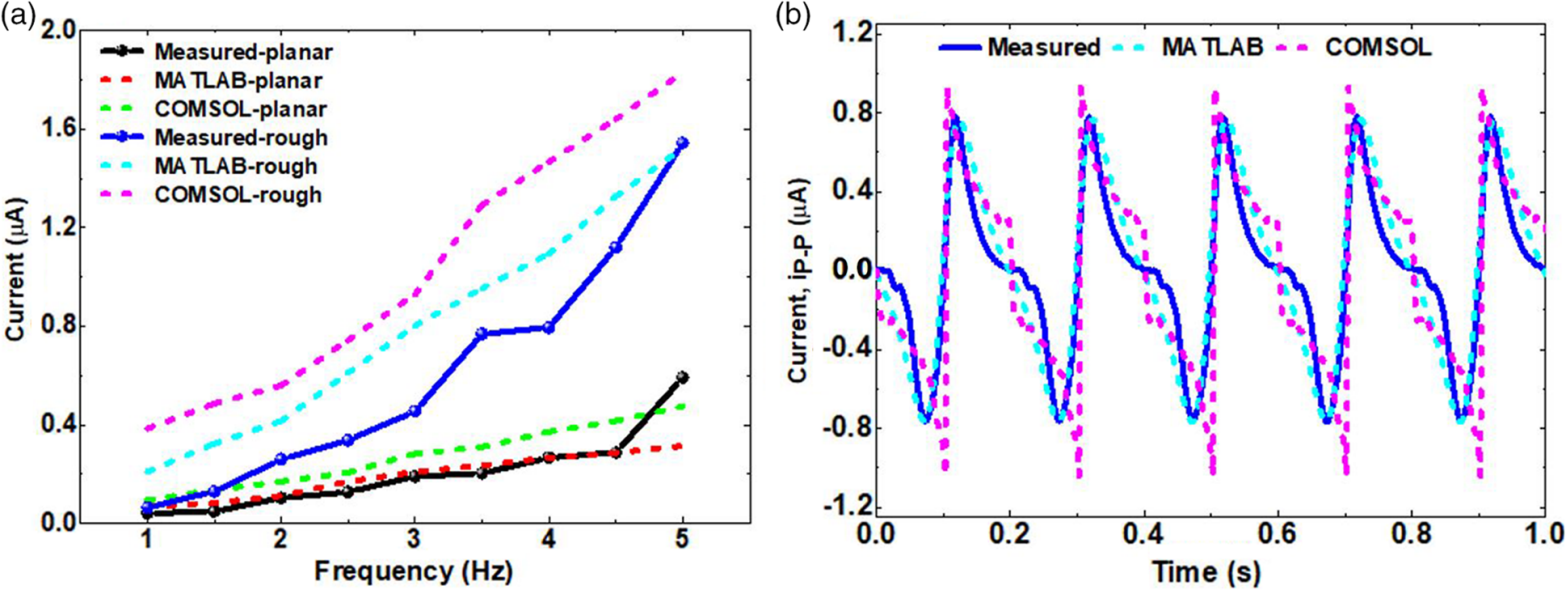
a) Current comparison of planar and rough electrodes for measured, MATLAB, and COMSOL models. b) Time scale current plot for measured, MATLAB, and COMSOL models at 5 Hz modulation frequency.

REWOD generated AC current was measured for a frequency range of 1–5 Hz with 0.5 Hz step size for both the rough and the planar electrodes using a Keithley 2400 Sourcemeter in combination with Keithley data acquisition software, Kickstart 2.0. AC peak‐to‐peak current for the given frequencies ranged from 41 to 591 nA for the planar electrode and 65 to 1542 nA for the rough electrode. As discussed in the previous section, this range of currents also resulted from using a 50 μL droplet of electrolyte (DI water) and 0.33 cm^2^ of electrode–electrolyte interfacial area. Measured currents at the given frequency range were validated with the results from the MATLAB and COMSOL models. Results show a close agreement between the measured and the modeled data. A standard deviation of 113 nA from a single frequency at 5.0 Hz using the measured, COMSOL, and MATLAB data for the planar electrode, and that of 140 nA for the rough electrode closely validate the agreement. **Figure** **10**a shows a summary result, both measured and modeled, for both planar and rough electrodes. The measured AC current generation at 5.0 Hz modulation frequency corresponds to current densities of 1.8 and 4.67 μA cm^−2^ for planar and rough electrodes, respectively. In accordance with the voltage versus frequency, the AC current also shows almost a linear relationship with frequency. Increasing oscillation frequency increases the dynamics of the electrical charge transfer per unit time during the REWOD process resulting in a higher current (*i = dQ/dt*). Figure 10b shows a representative AC current plot for 1 s of time at 5.0 Hz frequency involving measured as well as data from the COMSOL and the MATLAB models.

### 4.4 Power Density Calculation

Power is calculated using the root mean square (RMS) values of the voltage for both rough and planar electrodes. Power density can be calculated using Equation (15)

(15) $$P_{\text{RMS}}=\frac{V_{\text{RMS}}^{2}}{4Z_{L}A}$$

where *V*_RMS_ is the RMS voltage, *Z*_L_ is an optimal external load, and *A* is the electrode–electrolyte interfacial area. Referring to Equation (15), the maximum power can be harvested when equivalent impedance (|*Z*|) is matched with an optimal external load |*Z*_L_|. Power density for both rough and planar electrodes is calculated for all the frequencies used in this work and is tabulated in **Table** **2**. Results from the table show significant increase in power density from planar to rough electrodes, demonstrating the contribution of rough surface area to the power output. In addition, a much lower rough electrode impedance compared to that of the planar electrode contributed to a much higher power density from the rough electrode. The maximum power density at 5 Hz frequency for the rough electrodes is determined to be 3.18 μW cm^−2^ which is ≈4 times higher compared to the planar electrodes.

**Table 2:** Comparison of RMS power density between rough and planar electrodes based on the measured results

Frequency [Hz]	RMS power density [μW cm^−2^]—planar	RMS power density [μW cm^−2^]—rough	Power density increase [times]
1.0	0.19	0.41	2.2
1.5	0.28	0.68	2.4
2.0	0.35	0.76	2.2
2.5	0.36	1.00	2.8
3.0	0.49	1.11	2.3
3.5	0.65	1.39	2.1
4.0	0.63	1.91	3.0
4.5	0.72	2.59	3.6
5.0	0.83	3.18	3.8

A performance comparison of various performance parameters from this work with some of the prior works based on REWOD energy harvesting is summarized in **Table** **3**. The maximum power density from this work is very good considering no bias voltage was used in this work. Higher power is clearly possible when a bias voltage is applied but that conflicts with the eventual idea of a self‐powered sensor. Results from this work take a step forward in harvesting energy from human motion activities without bias voltage that can potentially power up wearable sensors for human health monitoring in real time.

**Table 3:** Power density comparison with prior works based on REWOD energy harvesting

References	Energy harvester	Frequency [Hz]	Power density [μW cm^−2^]	Bias [V]
Hsu et al.^[^ ^39^ ^]^	REWOD	300	10 000	4.5
Krupenkin et al.^[^ ^5^ ^]^	REWOD	2	10 000	60
Yang et al.^[^ ^17^ ^]^	REWOD	3	10 960	24
Huynh et al.^[^ ^40^ ^]^	REWOD	6	0.096	1.2
This work	REWOD	5	3.18	≈0

## 5 Conclusion

REWOD energy harvesting was performed using high surface area rough electrodes. It is validated through COMSOL and MATLAB modeling that the rough surface electrodes possess a much higher surface area and hence an increase in the effective electrode–electrolyte interfacial area. This results in a much higher power density output compared to that of the planar electrodes. REWOD‐generated AC current and AC voltage along with the resistance and capacitance for a frequency range of 1–5 Hz with 0.5 Hz step size were measured. A maximum power density output of 3.18 μW cm^−2^ from a 50 μL DI water droplet as an electrolyte at 5 Hz oscillation frequency is demonstrated which is ≈4 times higher than that of the planar electrodes. To better understand the effect of surface area on REWOD performance, MATLAB and COMSOL‐based mathematical models were developed and validated with measurement data. This work demonstrates that using high surface area electrodes such as rough surfaces will significantly enhance power density, potentially fully powering wearable motion sensors from human motion activities alone. As wearable motion sensors have been miniaturized over the years, implementation of high surface area electrodes would complement the trend toward miniaturization because of higher power density. This work also illustrates that REWOD has the potential to fully self‐power wearable sensors without the need for an external bias source.

## Conflict of Interest

The authors declare no conflict of interest.

## Supporting information

Supplementary Material

## Data Availability

The data that support the findings of this study are available from the corresponding author upon reasonable request.
