# Supplementary material for: Advancing Reverse Electrowetting‐on‐Dielectric from Planar to Rough Surface Electrodes for High Power Density Energy Harvesting
Source: Energy Technol (Weinh). 2022 Jan 7;10(3):2100867. doi: 10.1002/ente.202100867 (PMC9285574; doi:10.1002/ente.202100867)
Supplement: Supplementary file 1 — Supplementary Material [file ENTE-10-0-s001.pdf]

Supplementary Information

Advancing Reverse Electrowetting-on-Dielectric from Planar to Rough Surface Electrodes for High Power Density Energy Harvesting

Pashupati R. Adhikari[a, *], Adnan Patwary[b], Karthik Kakaraparty[b], Avinash Gunti[b], Russell C. Reid[c], Ifana Mahbub[b]

[a] Department of Mechanical Engineering, University of North Texas, Denton TX 76207

[b] Department of Electrical Engineering, University of North Texas, Denton, TX 76201

[c] Department of Engineering, Dixie State University, St. George, UT 84770

* Corresponding Author: Department of Mechanical Engineering, University of North Texas, 3940 N Elm St, Suite F101, Denton, TX 76207

Email address: pashupatiadhikari@my.unt.edu

Appendix A. Derivation of voltage and current equations in parallel arrangements of a resistor R_P, a capacitor $C_P(t)$, and a current source $I_P(t)$.

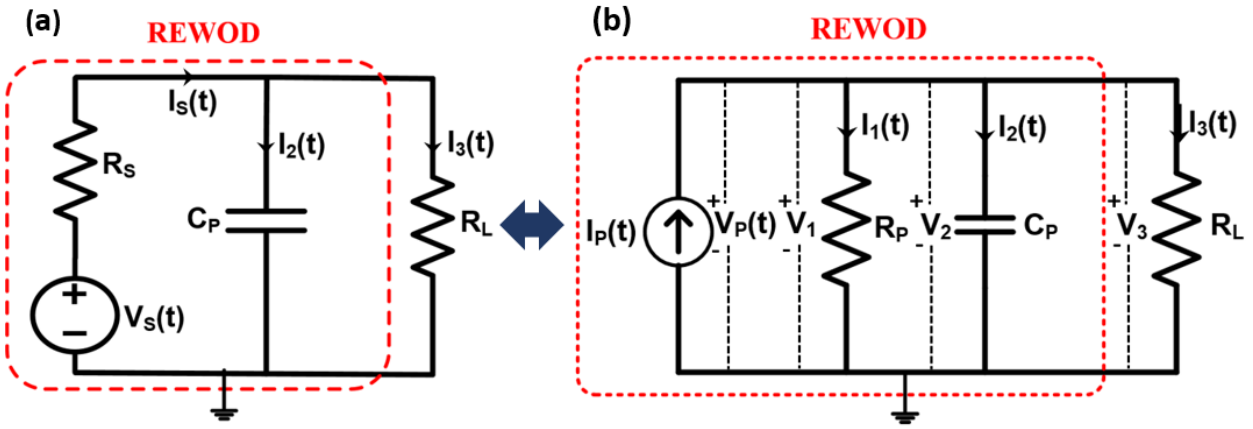

Figure A1. (a) Lumped element model of a REWOD energy harvester in series configuration. (b) Lumped element model of a REWOD energy harvester in parallel configuration. Series and parallel configurations are equivalent to one another.

In Figure A1(a), the source voltage $V_S(t)$ has two components: the DC component and an AC component. The DC component is due to leakage current which is always present even when there is no modulation. Therefore, the $V_S(t)$ can be expressed by Equation (A1):

$$V_s(t) = V_{S,DC} + V_{S,AC}(t) \tag{A1}$$

Similarly, the current passing through the capacitor (C_P), $I_2(t)$ is given by the partial derivation of $C_P(t)$ with respect to both the DC and AC components of $V_S(t)$ as shown in Equation (A2):

$$I_2(t) = V_{S,DC}\frac{\partial C_P(t)}{\partial t} + C_p(t)\frac{\partial V_{S,AC}(t)}{\partial t} \tag{A2}$$

Applying Kirchhoff's current law in the circuit diagram in Figure A1(b), $I_P(t)$ is determined as given by Equation (A3):

$$I_P(t) = I_1(t) + I_2(t) + I_3(t) \tag{\textbf{A3}}$$

Since the R_P, C_P, and R_L are all in parallel to each other in the circuit diagram, $V_P(t)$ is determined as given in Equation (A4):

$$V_P(t) = V_1(t) = V_2(t) = V_3(t) \tag{A4}$$

Since $V = IR$ (Ohm's law), $V_P(t) = I_1(t)R_P = I_2(t)X_{CP}(t) = I_3(t)R_L$

where $V_P(t) = I_P(t)\,Z_L(t)$; $Z_L(t) = R_P||X_{CP}(t)||R_L$ and $X_{CP}(t) = \frac{1}{2\pi f C_P(t)}$

By substitution, Equation (A4) is transformed into Equation (A5):

$$V_P(t) = \{I_1(t) + I_2(t) + I_3(t)\}Z_L(t) \tag{A5}$$

Converting all of the terms in the parenthesis to a function of $I_2(t)$ results in:

$$V_P(t) = I_2(t)\left\{\frac{X_{CP}(t)}{R_P} + 1 + \frac{X_{CP}(t)}{R_L}\right\} Z_L(t) \tag{A6}$$

Simplifying the $\left\{\frac{X_{CP}(t)}{R_P} + 1 + \frac{X_{CP}(t)}{R_L}\right\} Z_L(t)$ term where $Z_L(t) = R_P||X_{CP}(t)||R_L$:

$$\left\{\frac{X_{CP}(t)}{R_P} + 1 + \frac{X_{CP}(t)}{R_L}\right\} Z_L(t) = \left[\frac{X_{CP}(t)R_L + R_P R_L + X_{CP}(t)R_P}{R_P R_L}\right]\left[\frac{1}{\frac{1}{R_P} + \frac{1}{X_{CP}(t)} + \frac{1}{R_L}}\right]$$

$$= \left[\frac{X_{CP}(t)R_L + R_P R_L + X_{CP}(t)R_P}{R_P R_L}\right]\left[\frac{R_P X_{CP}(t) R_L}{X_{CP}(t)R_L + R_P R_L + X_{CP}(t)R_P}\right]$$

$$= X_{CP}(t)$$

Finally, by substitution, Equation (A6) is transformed into Equation (A7):

$$V_P(t) = I_2(t)X_{CP}(t) = \left[V_{S,DC}\frac{\partial C_P(t)}{\partial t} + C_p(t)\frac{\partial V_{S,AC}(t)}{\partial t}\right] X_{CP}(t)$$

$$V_P(t) = \frac{V_{S,DC}\frac{\partial C_P(t)}{\partial t} + C_p(t)\frac{\partial V_{S,AC}(t)}{\partial t}}{2\pi f C_P(t)} \tag{A7}$$

Equations **(A3)** for current modeling and Equation **(A7)** for voltage modeling are used in the MATLAB modeling section (3.2) of the manuscript.
